# Development of Optical Label-Free Biosensor Method in Detection of *Listeria monocytogenes* from Food

**DOI:** 10.3390/s23125570

**Published:** 2023-06-14

**Authors:** Ana Fernández Blanco, Manuel Hernández Pérez, Yolanda Moreno Trigos, Jorge García-Hernández

**Affiliations:** 1 Lumensia Sensors S.L., 46022 Valencia, Spain; 2 Centro Avanzado de Microbiología de Alimentos, Biotechnology Department, Universitat Politècnica de València, 46022 Valencia, Spain; mhernand@btc.upv.es (M.H.P.); jorgarhe@btc.upv.es (J.G.-H.); 3 Instituto de Ingeniería de Agua y del Medioambiente, Universitat Politècnica de València, 46022 Valencia, Spain

**Keywords:** *Listeria monocytogenes*, food safety, biosensor, nanophotonics, bioreceptor, optical transducers

## Abstract

The present work describes an alternative method for detecting and identifying *Listeria monocytogenes* in food samples by developing a nanophotonic biosensor containing bioreceptors and optical transducers. The development of photonic sensors for the detection of pathogens in the food industry involves the implementation of procedures for selecting probes against the antigens of interest and the functionalization of the sensor surfaces on which the said bioreceptors are located. As a previous step to functionalizing the biosensor, an immobilization control of these antibodies on silicon nitride surfaces was carried out to check the effectiveness of in plane immobilization. On the one hand, it was observed that a *Listeria monocytogenes*-specific polyclonal antibody has a greater binding capacity to the antigen at a wide range of concentrations. A *Listeria monocytogenes* monoclonal antibody is more specific and has a greater binding capacity only at low concentrations. An assay for evaluating selected antibodies against particular antigens of *Listeria monocytogenes* bacteria was designed to determine the binding specificity of each probe using the indirect ELISA detection technique. In addition, a validation method was established against the reference method for many replicates belonging to different batches of meat-detectable samples, with a medium and pre-enrichment time that allowed optimal recovery of the target microorganism. Moreover, no cross-reactivity with other nontarget bacteria was observed. Thus, this system is a simple, highly sensitive, and accurate platform for *L. monocytogenes* detection.

## 1. Introduction

*Listeria monocytogenes* constitutes an objective of maximum interest for the food industry due to its highest percentage of incidence concerning the food outbreaks it causes. Its isolation is generally carried out in foods of animal origin, mainly meat and dairy products [1]. Nevertheless, it can also be found in fresh products such as salads [2]. Foods ready for consumption are significantly affected by the presence of this pathogen since it can survive even extreme environmental conditions maintained in the production and storage of food (high salinity, low pH, and cooling temperatures) [3].

*L. monocytogenes* is regulated in ready-to-eat food to meet the strict food security criteria implemented in most countries [4]. The frequency of the bacterium’s occurrence in the case of foods that allow its growth is less than 0.04 CFU/gr, whereas it is 100 CFU/gr for those foods in which the bacteria should not survive within [3]. The European Commission establishes a limit of 100 colony-forming units (CFU) per gram or mL for ready-to-eat food products. In foods produced for risk groups, the absence of *L. monocytogenes* is required in 25 g or 25 mL [5].

To detect pathogenic microorganisms transmitted through food, pre-enrichment and/or specific enrichment methods are performed, followed by a solid-medium culture that confirms molecular and/or serological tests [6,7]. The horizontal method for detecting and enumerating *Listeria monocytogenes* and *Listeria* spp. consists of performing food sampling followed by the enrichment of the said food portion and additionally allowing it to grow in a selective solid medium [8]. Therefore, the analysis takes 4 days to conduct in the case of the presence of the microorganism. For this reason, in recent years, the main focus has been on developing molecular methods for the early detection of the microorganism. Thus, PCR is the primary molecular method used in food analysis today [9,10].

Different methods based on label-free and label sensors were developed to show the variable detection limits [11,12]. Biosensors have previously been used to detect pathogens in liquid and solid food samples. However, their low detection limits (LODs) mean they have yet to achieve as much as those obtained using the PCR method. In more recent studies, sensibilities similar to those obtained using PCR have been obtained on previously enriched cultures of *L. monocytogenes.* An immunomagnetic separation combined with fluorescent antibodies and subsequent detection using biosensors was carried out [10,13]. Because of this, there is great interest within the food industry to develop new methods that can provide a quick and highly sensitive response, as well as produce similar results to those obtained by IQCHECK and AFNOR in the detection of the microbial contamination of food in real time [9,10].

It is necessary to prove the specificity of the antibodies used as probes on immunosensors [14,15,16] under different conditions of the growth of target microorganisms to verify the expression of the antigen [17,18,19] so that different nonspecific unions can be addressed in different experimental phases [20]. Microarrays on which different antibodies are available can serve as detection platforms to simultaneously allow multiplex detection of several food pathogens [21,22,23,24].

It is possible to integrate microfluidic systems with immunosensors [25]. This means that it is possible to automate the detections by reducing the test time and preventing possible errors in the reading of the results, as well as reducing the complexity of using the equipment and the associated costs. Biosensors can be manufactured as a device that is easy to handle using laboratory operators as a lab-on-chip (LOC) device that permits the detection of bacteria using immunological techniques [26].

There are other non-fluorescence detection methods, such as the improved SPR (surface plasmon resonance) and EIS (electrical impedance spectroscopy) biochips, which are fast, easy to use, of a low cost, and able to simultaneously detect several targets with high sensitivity in situ [27,28]. There is currently great interest in developing lab-on-chip (LOC) technologies that reduce the complexity of use by using a smaller volume of samples and reagents, increasing the surface–volume ratio through fluidic microchannels. Additionally, there is the possibility of using complementary accessories such as microcapillaries, micropores, microfilters, and mixers [11]. Significant results have been achieved using magnetic beads attached to microbial antigens by successive magnetic fields at a determined frequency, achieving detections of bacteria with a sensitivity ranging from 10^2^ cells·mL^−1^ to very few bacteria [29]. In this sense, EIS protocols have allowed the detection of *L. monocytogenes* with low LODs of 1 log CFU/mL [29] and multiplex analysis [30]. However, more studies are necessary to determine this method’s reproducibility and robustness and prove that it applies to various food matrices.

In addition, developments have been made with optical sensors applicable to different areas of medicine and biology, detecting viruses and bacteria that are the objective of detection in food quality and safety checks [31,32,33]. Technology offers advantages relative to other methods, such as a lower detection limit, and it can differentiate *L. monocytogenes* from *Listeria* spp. that are not pathogenic to humans [34]. This work aims to develop and optimize a bio-photonic immunosensor to detect the presence of *L. monocytogenes* using functionalized ring resonators located on a silicon nitride surface. The method is based on the biofunctionalization of integrated photonic circuits (PICs) for the microbiological control of meat products, fresh vegetables, and ready meals (identification and quantification), allowing it to overcome the limitations of the current systems and techniques.

## 2. Materials and Methods

### 2.1. Bacterial Strains and Culture Conditions

The reference strains *Listeria monocytogenes* CECT936 (Spanish Type Culture Collection, Valencia, Spain), *L. innocua* CECT910, and *L. monocytogenes* M4 (isolated from a food matrix in a routine microbiological control in the food industry) were used for all assays. Bacteria were grown in Fraser Broth (Merck, Darmstadt, Germany), and plates were incubated at 30 °C for 3 h and 24 h in aerobic conditions as recommended.

### 2.2. Meat Samples

The response of the biosensor was also evaluated with several replicates of deep-frozen hamburger meat samples (25 g in 250 mL of Half-Fraser medium), which were inoculated with *L. monocytogenes* in a concentration range between 10^1^ and 10^10^ CFU/mL. Furthermore, different batches of frozen hamburger samples were collected over 1 year in a meat-processing facility in the Valencia area; they had already been evaluated by qPCR and plate-counted for the presence of *L. monocytogenes*, resulting positive. These samples were obtained directly from the packaged preparations and were ready for consumption.

### 2.3. Reagents and Antibodies

Polyclonal and monoclonal primary ELISA and array antibodies were evaluated against diverse target antigens from *L. monocytogenes*: rabbit polyclonal antibody anti-*Listeria* (Abcam) and mouse monoclonal antibody anti-*Listeria* (Invitrogen, TermoFisher Scientific, Waltham, MA, USA).

iELISA reagents included commercial TMB substrate (Thermo Scientific, Waltham, MA, USA), H_2_SO_4_ solution (slowing down the reaction), and 0.1 M of hydrochloric acid, Scharlab.

Functionalization reagents included CTES (carboxyethylsilanetriol, disodium salt 25% in MiliQ water, ABCR) 1%, EDC (1- Ethyl-3-(3-dimethylaminopropyl) carbodiimide, Sigma)/NHS (*N*-hydroxysuccinimide, Sigma), and MES (2-(N-morpholino)ethanesulfonic acid, ThermoFisher, Waltham, MA, USA) 0.1 M.

Secondary ELISA antibodies included GARPO polyclonal anti-rabbit IgG (whole molecule)–peroxidase antibody produced in goat, and GAMPO polyclonal anti-mouse IgG (whole molecule)–peroxidase antibody produced in goat.

Secondary array antibodies included GAM goat anti mouse IgG–Alexa 647 Abcam and GAR goat anti rabbit IgG–Alexa 647 Abcam.

Sensors probes included rabbit polyclonal antibody anti-*Listeria* (Abcam, Cambridge, UK) and rabbit polyclonal antibody anti-Fish (Eurofins Inmunolab, Reinbek, Germany) as a negative control.

### 2.4. Antibody Array or Immobilization Control Test

An antibody immobilization control was carried out specifically for *L. monocytogenes* on four flat silicon nitride surfaces. First, the surfaces were oxidized in 5 mL of HCl solution (0.1 M of hydrochloric acid, Scharlab, Barcelona, Spain) for 30 min in an orbital shaker plate at 30 rpm and were then washed with water and dried with an air current.

Next, each surface was immersed in 500 µL of CTES solution (carboxyethylsilanetriol, disodium salt 25% in water, ABCR) at 1%, capping the vial without closing and keeping it at room temperature for 2 h, shaking gently at 30 rpm. Then, the surfaces were washed with acidic (acetic acid) water and dried with air. Finally, they were dried in an oven incubator for 30 min at 110 °C, tempered in a desiccator for 10 min, and incubated at 4 °C overnight.

Surfaces 1 and 2 were immersed in 500 µL of a 2:1 molar ratio of EDC (1-Ethyl-3-(3-dimethylaminopropyl) carbodiimide, Sigma)/NHS (*N*-Hydroxysuccinimide, Sigma) in 0.1 M MES (2-(N-morpholino) ethanesulfonic acid, (10419123, ThermoFisher, Waltham, MA, USA), while surfaces 3 and 4 were not activated and were immersed in 500 µL of water at room temperature for 30 min. Finally, they were washed with 0.1 M MES and water, and then dried in air. A total of 0.8 µL was placed dropwise on each row of the surface. On all surfaces (S1, S2, S3, and S4), four drops of the specific monoclonal antibody for *L. monocytogenes* were added; in the second row, four drops of the polyclonal antibody were added; in the third row, four drops of PBS 1× was added as the negative control. In the fourth row of surface 1 (S1), 0.8 µL of monoclonal antibody was added, whereas, in the fourth row of surface 2 (S2), 0.8 µL of polyclonal antibody was added; the fourth row of the other two surfaces (S3 and S4) was left empty. These antibody solutions were made at a final concentration of 200 µg/mL in PBS 1×, and each surface was left under controlled humidity conditions for 2 h, after which all the surfaces were washed with PBS 1×. Finally, all the surfaces were blocked by submerging them in BSA 1% in PBS 1× and were incubated overnight at 4 °C in the refrigerator.

**Figure 1:**
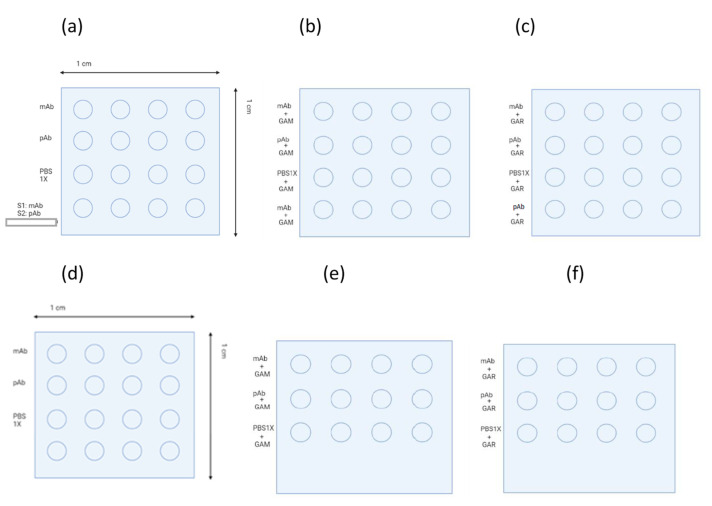
Graphic representation of the array layout. (**a**) S1 and S2 activated surfaces with primary antibody immobilization (lines 1,2), along with a negative (line 3) and a positive (line 4) control test. (**b**) S1 activated surface with primary and secondary antibody anti-mouse immobilization. (**c**) S2 activated surface with primary and secondary antibody anti-rabbit immobilization. (**d**) S3 and S4 nonactivated surface with primary antibody immobilization (lines 1,2) and negative control test (line 3). (**e**) S3 nonactivated surface with primary and secondary antibody anti-mouse immobilization. (**f**) S4 nonactivated surface with primary and secondary antibody anti-rabbit immobilization.

A standard solution of *L. monocytogenes* (included inside Transia AG *Listeria* 67000-96 kit) was used as a positive control. It was added only in the fourth row of surfaces 1 (S1) and 2 (S2), and then incubated at room temperature for 30 min. After 30 min, all the surfaces were washed with PBS 1× and water. Later, the conjugated antibody was added to all the rows and incubated at room temperature for 30 min. Afterward, all the surfaces were washed with PBS 1× and water, but they did not dry. In total, 50 µL of the GAM secondary antibody solution conjugated with a fluorophore (goat anti-mouse IgG–Alexa 647, Abcam) was added on surfaces 1 and 3 for the immobilization control of the monoclonal antibody, and then incubated for 1 h at room temperature in darkness. The surfaces were washed with PBS 1× and water. In the case of surfaces 2 and 4, 50 µL of the solution of the GAR secondary antibody conjugated with a fluorophore (goat anti-rabbit IgG–Alexa 647, Abcam) was added to control the immobilization of the polyclonal antibody, which was incubated and washed in the same way as in the case of surfaces 1 and 3. Control of the antigen–antibody binding reaction was performed on a flat surface, using the microarray technique by a direct assay, as shown in Figure 1.

Finally, the fluorescence intensity was measured with a GenePix 4000B Axon Instruments fluorescence microarray reader (Molecular Devices, LLC, San Jose, CA, USA, EE. UU).

### 2.5. iELISA (Indirect ELISA)

An indirect ELISA protocol was designed to evaluate the binding capacity. The protocol was based on the method used by Etty et al. [35].

#### Polyclonal Antibody Anti-Listeria iELISA

The polyclonal antibody (rabbit polyclonal antibody anti-*Listeria*, Abcam ab35132) was evaluated against different concentrations of the strains *L. monocytogenes* M4 and *L. monocytogenes* CECT936. Furthermore, it was also estimated against different replicates of deep-frozen hamburgers contaminated by *L. monocytogenes* in the food industry. All the samples were inoculated in duplicate in Semi-Fraser Broth. In addition, a PBS 1× solution was used as a negative control, and a standard solution of *L. monocytogenes* was used as a positive control.

A 96-well ELISA microplate was inoculated with 100 µL/well of the corresponding concentration of *L. monocytogenes* strains (M4 and CECT936), as well as different serial dilutions of the replicates of a batch of deep-frozen hamburgers contaminated by *L. monocytogenes* in the food industry to determine the specificity of the antibody. Then, the wells were washed three times with PBS 1× with 0.05% Tween-20. Subsequently, all the wells were blocked with 1% BSA in PBS 1× by adding 100 µL to each well of the plate and were incubated for 1 h at room temperature (25 °C).

After washing, 100 μL of a PBS 1× solution containing 1 ppm of the selected antibody specific for *L. monocytogenes* was added to all wells previously inoculated with antigen. All of the plates were incubated at 37 °C for 1 h, leaving a control without an immobilized antibody. Subsequently, three successive washes were carried out with PBST 1×, and the wells were filled with 100 μL of goat anti-rabbit–HRP conjugate, followed by incubation at 37 °C for 1 h. After carrying out three more washes with PBST, the detection reaction was carried out by adding 100 μL of substrate based on 1,2-diaminobenzene (OPD) (4 mg of 1,2-diaminobenzene and 15 μL of H_2_O_2_ in 10 mL of citrate buffer, pH 4.5). After waiting 15 min, the reaction was stopped by adding 50 μL of 2 M sulfuric acid. Finally, the emitted absorbance was read at 450 nm and 650 nm using a Varioskan Flash multimode spectral scanning plate reader (Multilabel Victor 1420 Counter). The positive control used was defined as a sample in which the OD450 nm value was 2.1 times higher than the negative control (P/N ≥ 2.1).

### 2.6. Optical PIC Functionalization

Silicon photonics has become the mainstream technology for photonic integrated circuits (PICs) [36,37]. An important reason is that, once the basic structures were optimized, the complete design of the PIC was transferred to the silicon nitride samples and wafers in large volumes at low cost using a CMOS-compatible fabrication process.

The manufacturing process of the Optical PICs designed for this work, which was carried out in a class 10–100 cleanroom environment, is based on a straightforward writing electron beam process on a positive resistance layer of polymethyl methacrylate (PMMA) with a thickness of 100 nm. After developing the resistance, a metal mask is produced by chromium evaporation after a peeling process. Such a metal mask is used to make the ion-etched inductively coupled reactive plasma (ICP-RIE) of the silicon nitride layer using fluoride gases.

**Figure 2:**
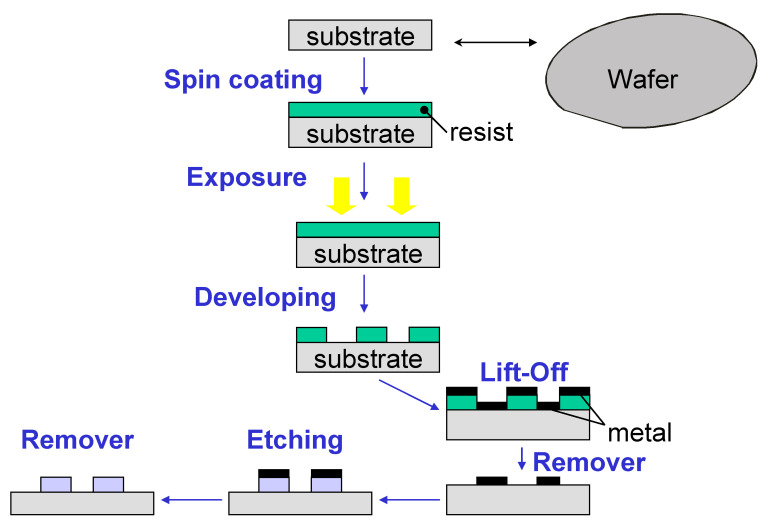
Schematics of the overall steps of the manufacturing process of Si_3_N_4_ PICs.

As shown in Figure 2, manufacturing begins with the deposit of a positive resin on the wafer. This resin is exposed to the circuit patterns using different lithography techniques (electron beam process or UltraViolet light). After development, the resin is used as a mask in an evaporation and chrome liftoff process. This chrome mask is used for the silicon nitride attack so that the protected zone is not attacked. Finally, the chromium is removed through a chemical process, such that the patterns are transferred to the sample or nitride wafer. Once the nitride guides have been etched, the silicon oxide layer covers the wafer. In this layer, windows have to be opened on the rings to perform the sensing so that the sample comes into contact with the rings functionalized with the corresponding antibodies.

In this case, a first exposure of a level of protection is carried out, metalized with chrome before the deposit of silicon oxide. Subsequently, a new exposure is made, and the oxide attack is carried out on the rings, creating the windows. Finally, the chrome is removed with a chemical process.

Therefore, after depositing a silicon dioxide layer (1 μm thick) by plasma-enhanced chemical vapor deposition (PECVD), a window is opened in the resonant rings, removing the silicon dioxide with a second electron beam lithography, resulting in a new ICP-RIE engraving.

**Figure 3:**
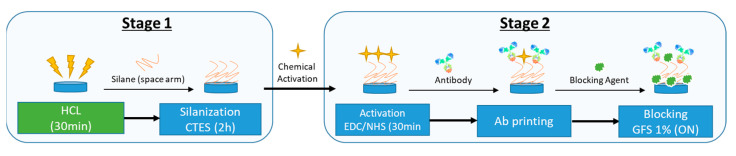
Graphic representation of functionalization PIC process.

A total of 100 photonic biosensors (PICs) from different batches/wafers were functionalized to detect *L. monocytogenes* (Figure 3). For starting the functionalization, the PIC surface was first oxidized by immersing in 5 mL of HCl solution (0.1 M of hydrochloric acid, Scharlab) for 30 min in an orbital shaker plate at 30 rpm. Subsequently, the surface was rinsed with deionized water (DIW) and dried. Later, silanization of the surface was carried out using a carboxyethylsilanetriol (CTES) solution for 2 h. In parallel, the activation of the carboxylic group of the CTES organosilane on the surface was carried out by adding a mixture of carbodiimide and *N*-hydroxysuccinimide (EDC/NHS) that was incubated for 30 min at room temperature. Subsequently, the surface was rinsed and dried in airflow. The rabbit polyclonal antibody anti-*Listeria* (Abcam ab35132), previously selected after obtaining the response of the developed iELISA, with higher specificity and sensitivity against *L. monocytogenes*, was covalently immobilized on the top in an oriented manner. Subsequently, the surface was rinsed with phosphate-buffered saline (PBS), dried again, and blocked with 1% GFS (gelatin from cold water fish skin) in PBS ON.

### 2.7. Microbiological Quantification

The enumeration of *L. monocytogenes* in food samples was performed according to EN ISO 11290-2: 1998 ⁄A1. 2004 (Anon. 2004b) with the use of ALOA medium.

Thus, a primary enrichment in half-Fraser Broth (Merck, Darmstadt, Germany) and a secondary enrichment in Fraser Broth (Merck, Darmstadt, Germany) were performed as described above. The cultures obtained from each enrichment step were plated on the selective agar media PALCAM and ALOA, incubated at 37 °C, and examined after 24–48 h for characteristic colonies. On the ALOA medium, the green–blue colonies surrounded by opaque halos were identified as *L. monocytogenes*, and the green–blue colonies without halos were identified as *L. innocua*. On the Rapid *L. monocytogenes* medium, the blue colonies were identified as *L. monocytogenes*, and the white ones were identified as *L. innocua*.

### 2.8. Detection Method for L. monocytogenes

Experimental assays were carried out to determine the optimal conditions of the alternative technique. Several exponential phase cultures (3 h) of *L. monocytogenes* (CECT936, M4) and *L. innocua* (CEC 910) were inoculated in Fraser Broth (Fraser Broth, Merck, Darmstadt, Germany), and meat samples were at four different concentrations of *Listeria monocytogenes*. They were then incubated for other times (3, 6, and 24 h) at 30 °C. Serial decimal dilutions counted the initial inoculum in 9 mL of Fraser Broth. All assays were performed in duplicate.

The samples described above in the ELISAS were tested in a photonic setup reader of PICs. Once the photonic chips were functionalized, attaching a microfluidic adhesive layer that allowed the sample to flow was necessary. For this experiment, a more superficial microfluidic layer (not the final microfluidic design to be used in the BACTERIO project) was bonded on the chip surfaces. The prepared photonic chips designed by the company Lumensia sensors were used for bacterial sensing using an optimized laboratory setup that uses a peristaltic pump. A flowing protocol was designed to perform the *L. monocytogenes* sensing experiment. First, a Fraser Broth solution was flowed for 3 min to obtain a reference signal. After that, the bacterial sample diluted in the same buffer was flown for 15 min. Finally, a cleaning buffer (Fraser Broth as in the first step) was flowed for 5 min.

Thus, to perform the initial validation of the detection technique, different *L. monocytogenes* sample dilutions were flowed on functionalized ring resonators at several dilution factors on Fraser Broth medium (Fraser Broth, Merck, Darmstadt, Germany). For the targets, *L. monocytogenes* CECT936, *L. innocua* CECT910, and *L. monocytogenes* M4, as well as different samples of the same batch of deep-frozen hamburgers naturally contaminated in the food industry *by L. monocytogenes* with a concentration range between 10^1^ and 10^10^ CFU/mL, were used.

### 2.9. Statistical Analysis

The plate counts obtained were expressed as the mean ± standard error of the duplicate samples of the numbers of colony-forming cells (CFUs) obtained and converted to log_10_. For each replicate of the experiment, different replicates of samples were analyzed to detect viable bacteria. In addition, each assay and subsequent biosensor detection were repeated at several weeks, using independently prepared *L. monocytogenes* inocula and different replicate samples from different batches of deep-frozen hamburgers. The effects of each variable were tested using an ANOVA test, and the differences in the frequencies of the positive samples were detected with a chi-square test at 95% significance, using Systat version 9 software (SPSS Inc., Chicago, IL, USA). USA). As determined via a one-way analysis of variance (ANOVA), the mean ± SD was statistically significant at *p*-values ≤ 0.05.

The specificity and sensitivity of the technique were demonstrated through a double-blind assay where negative frozen hamburger samples were contaminated with *L. monocytogenes* CECT936 and *L. innocua* CECT910. The results were analyzed for statistical significance [38]. The reproducibility of the technique was evaluated utilizing at least two repetitions of the detection, using a biosensor, of each of the concentrations of the study strains with the same handling conditions, instruments, and reagents.

For Antibody Array or Immobilization Control Test, a unifactorial ANOVA was performed. It evaluated whether statistically significant differences existed between the fluorescence intensity values of the activated surfaces and the non-activated surfaces.

## 3. Results and Discussion

Traditionally, the identification of *L. monocytogenes* involves using culture methods based on selective enrichment and seeding, followed by a characterization according to the colony’s morphology, the sugar’s fermentation, and the hemolytic properties. However, in recent years, the methods for detecting food pathogens have been more based on cultures, seeking to reduce the detection time. It is usually achieved by substituting the selective and differential steps using faster immunological or molecular assays. Rapid detection technologies are generally grouped into three categories: underlying technologies immunology, nucleic acid-based assays, and biosensors [39,40]. Specifically, the most widely used rapid tests are the enzyme-linked immunosorbent assay (ELISA), lateral flow immunoassay, and PCR [41,42].

### 3.1. Antibody Microarray Detection of L. monocytogenes

Characterizing biological probes or possible bioreceptors used on the biosensor based on the nanophotonic technology developed in this work is crucial to detect microorganisms in food matrices. These nanophotonic sensors are structures capable of guiding light, i.e., confining it. Upon laser excitation, if the analyte binds to the bioreceptor immobilized on the ring surface, there is a change in the refractive index, which, in that resonance, modifies the light guide in a way that is proportional to the amount of matter on the surface [43]. It is necessary to have specific recognition elements on the material’s cover for the analyte to be detected, making this technology selective. The choice of these probes is not trivial and requires characterization and optimization work, which is the first necessary step for developing a biosensor based on nanophotonic technology.

To verify the usefulness of biosensors concerning target detection, it is necessary to previously test the specificity of the antibodies that will be deposited as probes [14]. In addition, it should be verified how the selected target antigen reacts under different microbial conditions [17,19,44] to consider nonspecific interactions that may occur during the experimentation process [20,45].

The microarrays obtained on sensor surfaces where antibodies have been deposited as probes can be used as detection platforms [21,22,23,24]. This type of method has been shown to have a high level of multiplexing when applied to identify different pathogens in food. These antibodies used for detection can be marked with other fluorescent compounds [46,47] so that the fluorescence results can be read by employing laser scanners that allow the interpretation and quantification of the fluorescence signal obtained after exciting these molecules.

**Figure 4:**
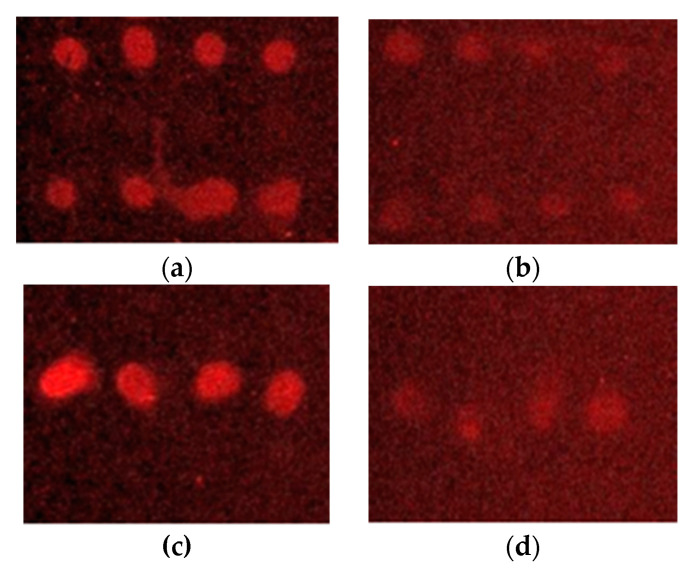
Images of silicon nitride surfaces after immobilization obtained by microarray fluorescence reader GenePix 4000B Axon Instruments. (**a**) The mAb activated surface S1 reacted with secondary anti-mouse antibody (line 1) and a serial dilution of an ELISA-positive control of *L. monocytogenes* (line 4). (**b**) The mAb nonactivated surface (S3) reacted insignificantly with the secondary anti-mouse antibody (line 1). (**c**) The pAb-activated surface S2 reacted with a secondary anti-rabbit antibody (line 2). (**d**) The pAb nonactivated surface (S4) reacted insignificantly with secondary anti-rabbit antibody (line 2).

**Figure 5:**
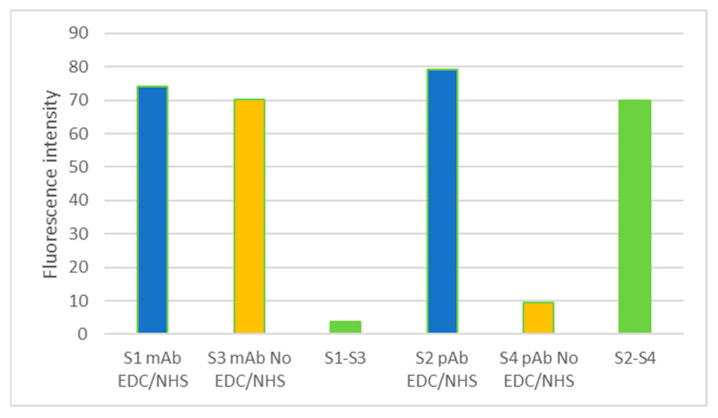
Fluorescence intensity results obtained for each surface activated and not activated by EDC/NHS, on which the pAb and mAb antibodies against *L. monocytogenes* were immobilized.

After immobilization of *L. monocytogenes* on silicon nitride surfaces, as expected, on the first surface in Figure 4a, we can observe the first four drops (line 1) corresponding to the immobilization of the primary monoclonal antibody, which reacted by joining the secondary anti-mouse antibody. The four drops of the last row (line 4) also correspond to the reaction of a serial dilution of an ELISA-positive control of *L. monocytogenes* bound to the immobilized monoclonal primary antibody. This result is in line with what was expected since the secondary antibody used was the monoclonal GAM (goat anti-mouse Alexa Fluor 647); thus, this secondary antibody was only bound to the monoclonal antibodies and did not bind to the immobilized polyclonal antibody immobilized on the same surface. In the same way, on surface 2 (Figure 4c), the four drops of the second row corresponding to the immobilization of the polyclonal antibody can be observed, having added the secondary antibody GAR (goat anti-rabbit Alexa Fluor 647). However, surfaces 3 (Figure 4b) and 4 (Figure 4d), which were not activated, showed a lower fluorescence intensity than those that were activated (Figure 5).

The fluorescence signal obtained for both covalently immobilized antibodies (pAb and mAb) by EDC/NHS activation on surfaces S1 and S2 was much higher than that obtained for the non-absorption-specific control of surfaces S3 and S4 (no EDC/NHS) (Figure 5). The result of the fluorescence intensity measurements of both antibodies on the activated surfaces (S1 and S2) corresponds to the component of the purely covalent bond formed between the antibody and the chemically treated surface. Therefore, it can be said that this immobilization control assay of both antibodies allowed us to verify that both were covalently bound to the chemically treated surface of the PICs to be used as sensor surfaces. Better results were obtained regarding the fluorescence intensity and covalent contribution of the chosen polyclonal antibody against *L. monocytogenes*.

A unifactorial ANOVA was carried out to evaluate if there were statistically significant differences between the fluorescence intensity values of the activated surfaces and nonactivated surfaces. In the case of surfaces with the immobilized monoclonal antibody, the statistically significant differences were perceived to be substantial if there was a *p*-value of 0.0018 (lower than the alpha value of 0.05), and if the value of the F statistic was more significant than the value of F from the tables. In the case of surfaces 2 and 4 with the immobilized polyclonal antibody, the differences were also observed to be statistically significant, with a *p*-value of 0.036 and an F value slightly more effective than the value of F in the tables. Lastly, a one-way ANOVA was performed to assess whether there were statistically significant differences between the monoclonal antibody and polyclonal antibody fluorescence values from the surfaces activated. No differences were observed in this case since there was a *p*-value of 0.38.

### 3.2. iELISA for the Detection of L. monocytogenes and Sensitivity Studies

ELISA provides a sensitive and selective method for detecting various antigens in different matrices [48,49]. One of the simplest realizations of an ELISA involves the immobilization of a target antigen on a plastic substrate by adsorption, and its detection with a selective antibody conjugated to a measurable label [50]. The resulting data provide a practical numerical value for quantitatively determining antigens in a sample and evaluating relative antigen concentration differences between samples. One advantage of an indirect binding ELISA (iELISA) is the requirement of only a single antigen and a specific antibody, as compared to the more sensitive capture ELISA (cELISA), which requires a pair of antibodies: one to capture the native antigen from solution and the other which is used as a detector [51].

About the ELISA method to detect *L. monocytogenes*, although there are several immobilization options, the most common is that anti-*Listeria* antibodies are immobilized on a plate to capture *Listeria* antigens and subsequently bind primary antibodies and then secondary antibodies linked to an enzyme, or directly bind to a conjugated antibody. This method allows results to be obtained in approximately 3–5 h, making it significantly faster than culture techniques even though it is not as sensitive [52]. However, this research focuses on the characterization of antibodies that will be selected to be incorporated for developing a biosensor using an indirect ELISA in which the antigens are immobilized.

An indirect ELISA test protocol was used to evaluate the binding capacity of the *L. monocytogenes*-specific polyclonal and monoclonal antibodies to characterize which will be selected as a probe on the biosensor under development. This iELISA was designed explicitly following the methods used by several authors and after reviewing the existing literature on detecting this bacterium using different ELISA methods [35].

With the absorbance values obtained in the iELISA methods, calibration lines were received concerning the quantification values of the dilutions of *L. monocytogenes* strains (CECT936 and M4) used in this study, as well as against the concentration values in plate count terms obtained for *L. monocytogenes* in the frozen hamburger food matrix naturally contaminated by the said target.

The detection and quantification values of the different dilutions used (dilutions of the *L. monocytogenes* strains and the dilutions of the naturally contaminated meat food samples) were provided by the microbiology laboratories of the Polytechnic University of Valencia, performing seeding and plate quantification of an aliquot of each of the dilutions used in iELISA. For each result, the absorbance versus the concentration in CFU converted to log_10_ of *L. monocytogenes* was obtained for the commercial strain, wild strain, and hamburger contaminated by *L. monocytogenes*. Three statistically significant calibration curves were obtained after performing the iELISA method to assess the sensitivity of the polyclonal antibody against *L. monocytogenes* selected as a probe of the biosensor studied here.

**Figure 6:**
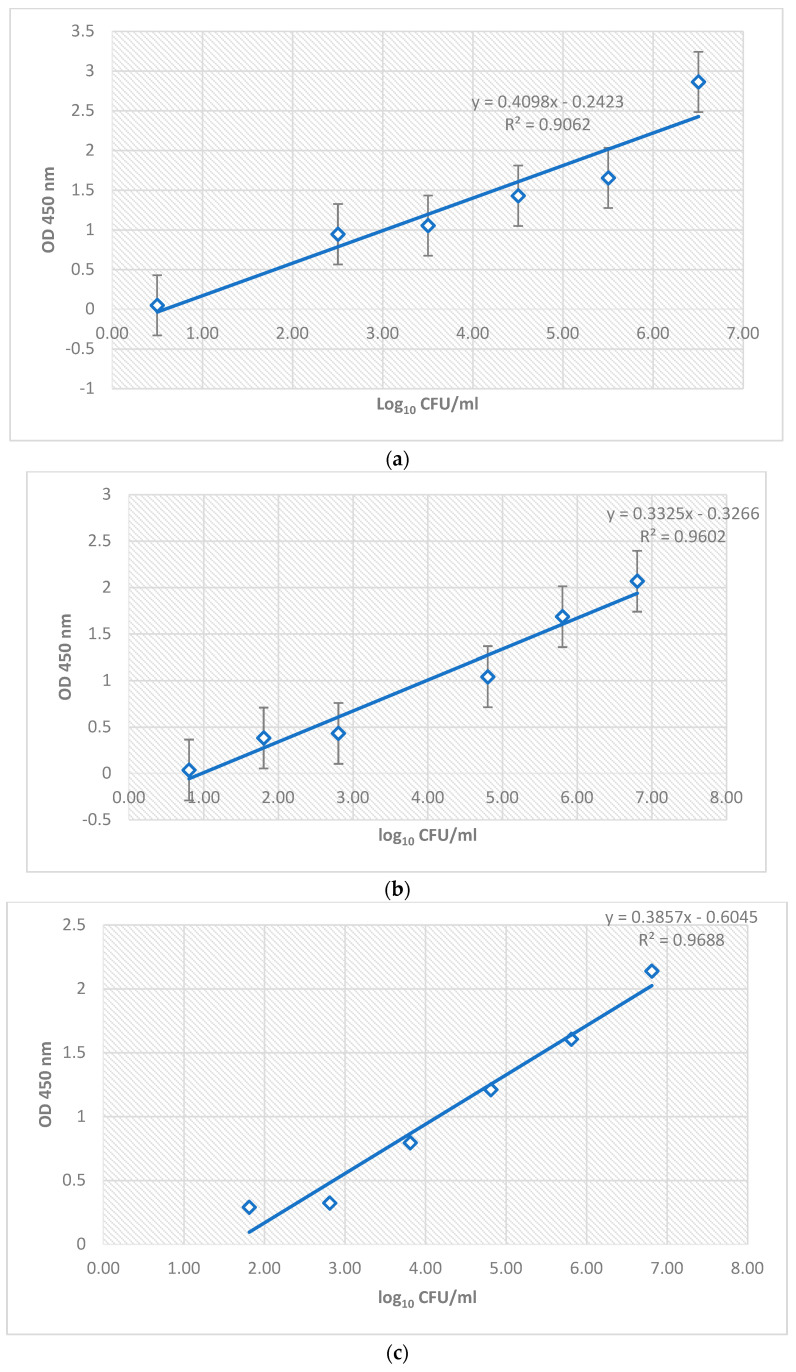
Calibration curves were obtained after relating the absorbance values (OD 450 nm) versus bacterial concentration in log CFU/mL obtained for iELISA anti-*Listeria* polyclonal antibody, along with its equation and R^2^ value. (**a**) Calibration curve obtained for five increasing dilutions of wild strain M4 of *L. monocytogenes* against the anti-*Listeria* polyclonal antibody. (**b**) Calibration curve for five increasing dilutions of commercial strain *L. monocytogenes* CECT936 against the anti-*Listeria* polyclonal antibody. (**c**) Calibration curve for five increasing dilutions of the hamburger contaminated by *L. monocytogenes* against the anti-*L. monocytogenes* polyclonal antibody.

As can be observed in Figure 6a, there was a relationship between the absorbances and the value of log CFU/mL with a correlation coefficient R^2^ of 0.9062. A higher concentration of the M4 strain led to a higher absorbance value observed; therefore, this was related to a correct binding of the antigen to the plate and a higher binding efficiency of the polyclonal antibody used against this strain of *L. monocytogenes*.

Regarding strain CECT936 (Figure 6b), the correlation between the values was higher (R^2^ of 0.9602). However, the same trend can be observed in Figure 6a, whereby a higher log value CFU/mL led to a higher absorbance value.

The values obtained for the natural contamination of *L. monocytogenes* from the dilutions of the deep-frozen hamburger sample (Figure 6c) were statistically significant since the relationship established that a higher concentration of the target microorganism led to higher absorbance results, with a correlation coefficient of the curve of 0.9688.

The highest values of log CFU/mL coincide with the highest absorbance values in the curve obtained in Figure 6c; thus, the results are consistent with the previous bibliography studied [52,53,54]. Optimal results at both high and low concentrations of *L. monocytogenes* were observed. Therefore, it could be concluded that this polyclonal antibody does not saturate at high concentrations [54] and has a high binding efficiency at both low and high concentrations of the *L. monocytogenes* antigen.

### 3.3. Immunosensor Detection of L. monocytogenes

Biosensors are analytical detectors consisting primarily of four elements: bioreceptor, transducer, sensor, and processor. The first is a biological recognition element (antibody, enzyme, etc.) whose function is to recognize the analyte of interest with the highest possible specificity. The bioreceptor is attached to a transducer and modifies the response when a union occurs between the analyte and the bioreceptor. This transducer converts the information into a quantifiable signal through the detector and sends it to a processor [55]. The main classification of biosensors is based on the transduction system used and the existing optical, electrochemical, thermal, and piezoelectric methods. An example of an optical transducer is a resonant ring sensor. In these transducers, there is a change in the refractive index of light when the analyte of interest binds to the bioreceptor immobilized on the surface of the ring. Thus, it is possible to relate the analyte concentration attached to the ring with the detected signal. Given the characteristics of the biosensors with optical transduction systems, this highlights the possibility of using much smaller volumes thanks to the fact that biosensors can integrate microfluidic and nanophotonic systems, thus permitting an analysis of the nanometric or micrometric system. In addition, another advantage is that the detection is carried out without labeling, monitoring measurements in real-time, and simplifying the number of steps and reagents.

Nanophotonic sensors are structures capable of guiding light, i.e., confining it. Upon laser excitation, each ring resonates at one wavelength and confines light in the ring. In contrast, when the binding of the analyte to the bioreceptor is immobilized on the surface of the ring, a change in the refractive index occurs and, in that resonance, the guidance should be modified in a way that is proportional to the amount of matter on the surface [43]. It must be arranged on the surface of the material recognition elements specific to the analyte to be detected to make this technology selective. The choice of these probes is not trivial. It requires a great deal of characterization work and optimization, this being the first necessary step for the development of a biosensor based on nanophotonic technology.

The detection used in this case is based on the phenomenon of photon transduction, which uses resonant cavities; specifically, it uses ring resonators made with silicon nitride technology. The study of the refractive indices obtained from the said ring resonators (RRs) has been proposed for multiple applications. Evanescent microring waveguide resonators have been demonstrated and fabricated using technical processes that are fully compatible with CMOS fabrication [55,56].

Resonant structures such as ring resonators (RRs) are suitable, but they need suitable interfaces with fiber optics to test them on wafers and use them in real applications. One of the advantages incorporated by this sensor regarding the light coupling scheme is the entire coupling system, which is carried out in free space. Thus, the collimator is not directly attached to the input gratings, being not necessary to connect a fiber to the PIC to allow the connection to the optical laser. The interface that satisfies the above two requirements is the grating coupler (GC).

**Figure 7:**
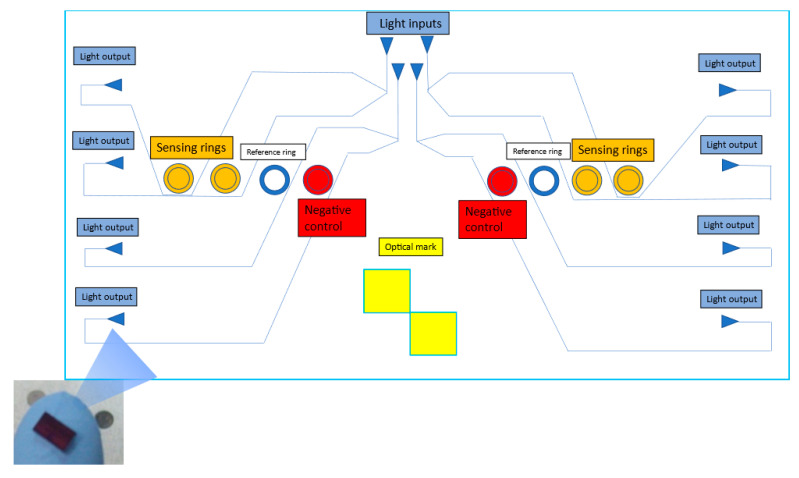
General schematic view of a PIC device. Detail of resonating structures (ring resonators with radius ~100 μm) used in the detection. In each circuit, eight rings are shown: four rings spread over two sensing areas that allow the detection of *L. monocytogenes* (sensing rings), two more rings (one per sensing area) to compensate for derivatives, and two additional rings as a negative control. These negative control rings bind to an analyte different from the objective, which will not be found in the matrix to be analyzed. In this case, the antibody deposited was an anti-fish antibody. The molecular receptors are represented in different colors to illustrate the multiple target capabilities of the technology. The input and respective light outputs in each channel are also indicated on the scheme, divided into four per detection channel or sensing area. The optical mark recognized by the developed hardware and software allows locating the PIC’s center, which helps the collimator focus the light on the input grating couplers. In addition, the optical mark allows presetting of the reading system. When the detection system recognizes it, it aligns the photodetector outputs with the laser so that the reading system captures the light from the output grating couplers.

The biosensor (PIC) designed for this study comprises three main elements: a detection ring resonator, a light coupling block (grating couplers), and an optical power distribution block. The lightly coupled section allows the introduction and pull of the optical signals in/out of the PIC, while the power distribution block is needed to feed all the ring resonators of the PIC with a standard laser source input. The detection system based on the optical laser moves or aligns it, helped by the visual marks indicated in Figure 6. Thus, the laser is aligned with the output photodetectors and the input collimator, as indicated in the legend of Figure 7.

Another advantage that the development of this biosensor provides concerning current detection systems is the multiplex ring system. The structure or design allows the simultaneous measurement of several targets of interest in a single measure since each ring resonator can be with a different probe. This fact represents a novelty compared to traditional systems (PCR, ELISA, etc.), where each target usually has to be analyzed separately. The eight ring resonators that make up the sensor are distributed over two channels or sensing areas composed of four ring resonators. This distribution allows there to include two detection replicas on which the same antibody is deposited, allowing it to bind to the *Listeria monocytogenes* bacterium, as well as two more rings that serve as a negative control (another probe, anti-fish antibody, whose binding antigen is different from the target bacterium and is not routinely detected in the food matrices under study) and a ring resonator that works as a blank (reference) since no probe is functionalized on it (Figure 7).

The sensitivity of waveguide sensors depends on the extent of overlap of the evanescent field with the sample to be analyzed [57,58]. Sensitivity is directly related to sensing parameters such as the limit of detection (LoD) and limit of quantification (LOQ). In this sense, the selected ring resonators showed sensitivities around 265 nm/RIU with quality factors of around 25,000. The sensors used in this study work with TE-polarized light. The TE predominance also occurs in the 15,500 nm wavelength window for SiN [59] when operating the system in TM mode. When characterizing said biosensor under development, the studies carried out in parallel allowed us to conclude that it could also run the system at TM mode, which could mean greater detection sensitivity. The surface sensitivity of these biosensors could be increased by using another wavelength, i.e., 1310 nm [60].

Photonic biosensors implemented on silicon PICs enable label-free performance with high detection sensitivities, and they are ultimately disposable, which is remarkably interesting for point-of-care diagnostics without requiring specialized personnel [61]. The present detection system, developed to allow the capture of the transduction signal emitted by the sensor when passing the analyzed samples, is based on the union of three elements that constitute a measurement setup. A PIC (designed by the company Lumensia sensors), a two-channel microfluidic system coupled to said PIC, and a peristaltic pump on which both microfluidic channels are connected to the microfluidic system attached to the PIC were fixed, over which the samples to be detected would pass.

**Figure 8:**
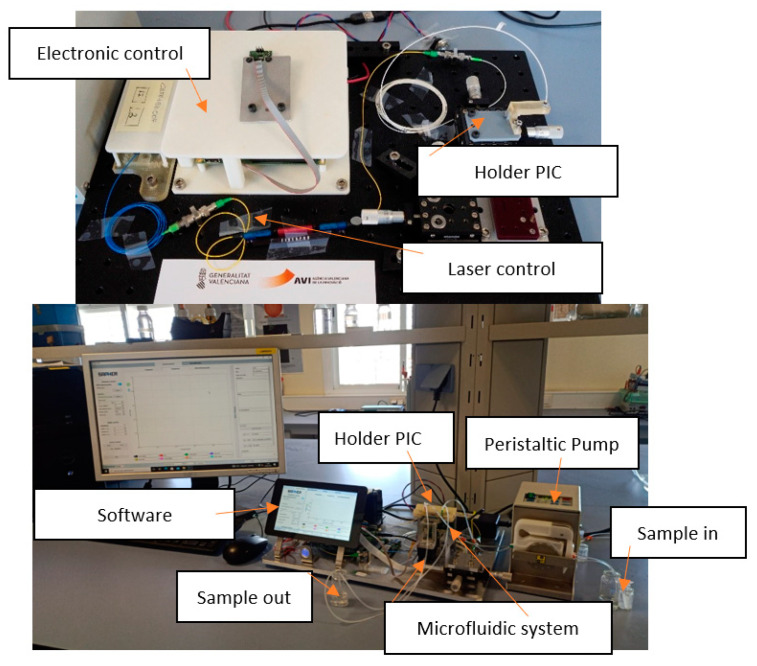
Photonic setup reader and measurement equipment for PICs or photonic sensors.

There was also a setup for reading the data obtained in signal transduction regarding resonance (Figure 8). This reading system is based on software and hardware designed by the company Lumensia sensors, allowing the optical signal to be translated into resonance measured in pm.

A flowing protocol was designed to perform the *L. monocytogenes* sensing experiment. First, a Fraser Broth solution was flowed for 3 min to obtain a reference signal. After that, the bacterial sample diluted in the same buffer was flown for 15 min. Finally, a cleaning buffer (Fraser Broth as in the first step) was passed for 5 min.

The integration of the entire detection system (photonic biosensor, coupled microfluidics, electronic module, and measurement software) is an additional novelty since, up to now, the food pathogen studied (*L. monocytogenes*) has yet to be detected in this way. That is, the originality of this work is found in the detection application. This integrated system makes it possible to capture the change in the light response when the specific binding of the immobilized antibody on the sensor surface occurs, which does not need to be labeled with a fluorophore against the target antigen (particular protein or enzyme on the surface of the *L. monocytogenes*).

Today’s trend in biosensors is simplifying the detection process to improve accessibility and robustness. Thus, the contribution of the coupled microfluidic system allows the simultaneous measurement of two samples at the same time since the eight ring resonators that make up the sensor are distributed over two channels made up of four resonant rings, enabling sensitivities down to a ng/mL scale with response times <30 min.

**Figure 9:**
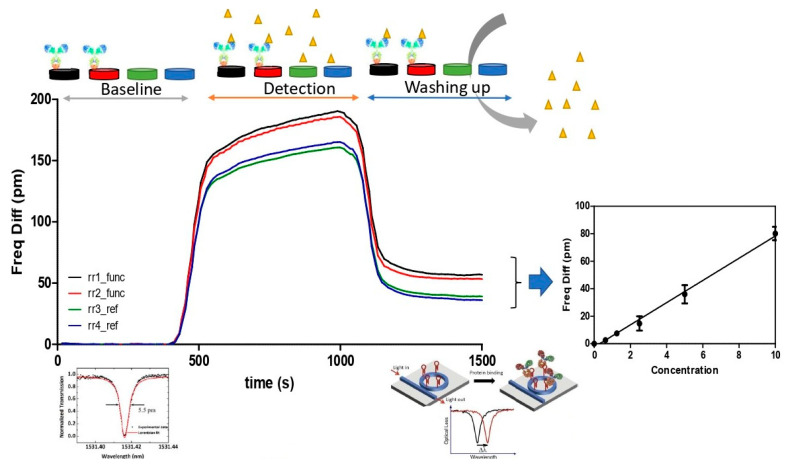
Schematic representation of sensogram and acquisition of data obtained by functionalized photonic integrated circuit (PIC) surface measurement. The average flow of a contaminated sample from its initial washing is shown (to establish a baseline), along with the flow of the contaminated sample in which the binding of *Listeria monocytogenes* antigen to antibody fixed on the surface of the PIC (Detection) occurs, and the final washing process of the PIC surface to remove unwanted residues. The correlation between resonance in pm and time in s in a flow of a sample contaminated by *Listeria monocytogenes* is shown. On the right, the translation of the optical signal performed by the system is shown in the form of an internal calibration curve in which the resonance in pm is correlated with the concentration of bacteria obtained by subtracting the net difference obtained between the resonance obtained by the reference rings and the functionalized rings (rr1 func, rr2 func, rings functionalized with the antibody against the target bacteria, rr3 ref negative control reference, rr4 ref blank control reference).

The reading software designed by Lumensia sensors allows the translation of the optical signal obtained in the form of an evanescent wave and receiving a measurement sensogram (Figure 9), in which the optical response for the resonance obtained after a first wash of the sensor or PIC with the buffer can be reflected off the passage of the sample (or antigen-immobilized antibody union), and a second wash captures the difference in the optical response after undoing the antigen-antibody union. All of this is represented as a measurement sensogram relating the obtained resonance (*Y*-axis) to time in seconds (*X*-axis). As defined in the figure provided, the positive difference obtained in terms of resonance between the rings functionalized with the antibody against the bacterial target concerning the reference ring resonators (negative control and blank) provides a resonance value in pm, which the software collects as a positive result if it detects the presence of the antigen of the bacterium under study. Thus, quantitatively and indirectly, it permits an estimation of the transfer of the amount of CFU/mL of the target in the sample of interest.

Plate counting of *L. monocytogenes* carried out for each experiment confirmed the efficiency of the immunosensor method in detecting *L. monocytogenes* in a presence/absence response test (Table 1) with a range of 10^2^ to 10^6^ CFU/mL orders of magnitude in the immuno-separated cell suspension.

To test the immunosensor and the enrichment method that most efficiently detected *Listeria monocytogenes*, various strains of *L. monocytogenes* and *L. innocua*, as well as fresh natural contaminated meat samples (deep-frozen hamburgers,) were used. All the samples used and their respective detection results, which permit an evaluation of the efficiency of the immunosensor-based detection, are labeled in Table 1.

The limit of detection (LoD) analysis found in deep-frozen hamburgers using the said biosensor after 3 h of enrichment reported a detection rate of 100% for 10^2^ CFU/mL of *L. monocytogenes* (*p* = 0.0026). The data obtained after the analysis by the biosensor method versus the traditional method, with a 95% confidence interval, demonstrated a relative accuracy or observed agreement, operating characteristics of sensitivity and specificity, and positive and negative predictive values of 100%. The sensitivity results were similar since only the samples contaminated with *Listeria monocytogenes* obtained a direct detection result using the developed biosensor. Samples without the microorganism or with an interfering microorganism such as *L. innocua* were not detected, revealing the technique’s specificity to detect true negatives and true positives for the presence of the microorganism [62]. The observed agreement or relative accuracy obtained for the alternative method indicated a 100% agreement between the response obtained by the reference method and the response received by the biosensor with identical samples. The positive predictive value obtained using a biosensor indicated 100% reliability for detecting positive samples. Likewise, the negative predictive value obtained for this method indicated a 100% reliability of detecting negative samples in deep-frozen hamburger samples.

These results show that the detection of *L. monocytogenes* from meat can be achieved with any of the two tested methods since they share the same specificity. The results were highly reproducible since detections by the specific binding of *L. monocytogenes* species antigens were observed in 96.8% of the contaminated samples, indicating a probability of 96.8% to find the same result among identical samples analyzed at different times under standard reproducibility conditions. The results of the reproducibility tests were obtained with the same detection method, on the same sample, with other operators, and with different measurement equipment.

**Figure 10:**
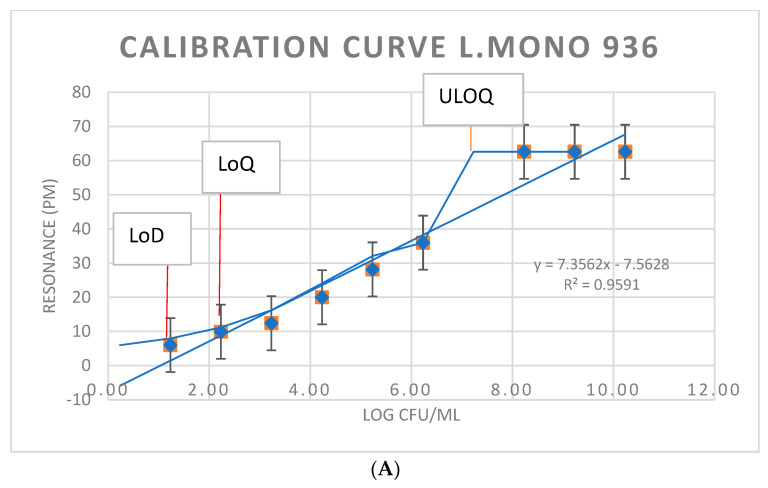

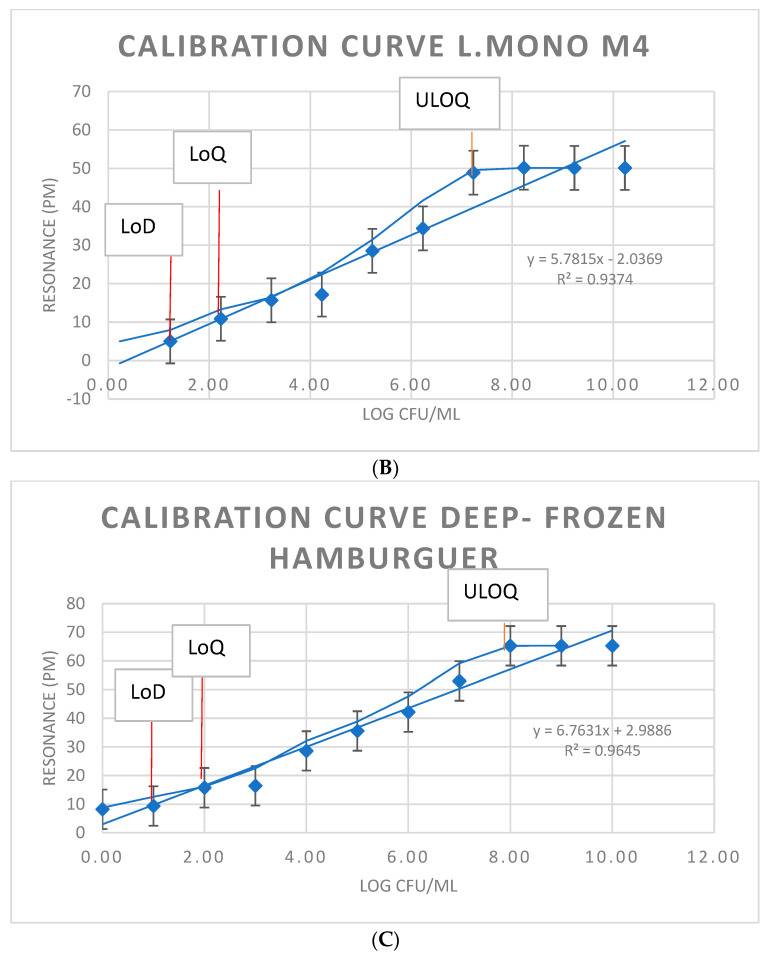
Calibration curves against commercial/wild strains of *L. monocytogenes* and meat matrix naturally contaminated by *L. monocytogenes*. (**A**) Calibration curve of biosensor against *L. monocytogenes* CECT936. (**B**) Calibration curve of biosensor against *L. monocytogenes* M4. (**C**) Biosensor calibration curve against *L. monocytogenes* isolated from naturally contaminated deep-frozen hamburgers. Perform at least six determinations (preferably 10) of samples at the calculated breakpoint concentration to estimate the baseline or threshold spread s0. The corresponding CFU/mL of *L. monocytogenes* in the enrichment cultures was derived from Table 1. The resonance in pm is the unit of measurement obtained by the laboratory setup reader used after inserting and flowing the contaminated samples shown in Table 1. LoD (limit of detection) calculated as LoD = 3.3 s0. LoQ (limit of quantification) calculated as LoQ = 10 s0. ULOQ, upper limit of quantification.

Figure 10 shows the photonic measurement responses (in terms of microring resonance notch shift–resonance in pm) obtained during the experiment against *L. monocytogenes* for different dilution factors (from 10^1^ to 10^10^ in Fraser Broth buffer). As can be seen, positive detection was achieved for up to 10^1^ dilution factors. It can also be observed that there was a clear dependence of the achieved results on the bacterial concentration. Thus, a less intensive optical signal was obtained for the more diluted sample.

The number of *L. monocytogenes* microorganisms in food samples is low (<10^2^ CFU/gr). However, in foods with a long refrigeration time, such as raw beef and chicken, the number of *L. monocytogenes* cells can be increased to 10^6^–10^8^ CFU/g of food [63,64,65]. For this reason, in the present study, we also replicated artificially contaminated samples of deep-frozen hamburger using reference *L. monocytogenes* strains and strains isolated from production environments around the said concentration range (M4).

The quantification of *L. monocytogenes* (CFU/mL) in the enrichment cultures was based on the calibration curve shown in Figure 10. The immunosensor method was also evaluated for the potential to provide a quantitative response for those bacterial pathogens that need to be enumerated for the definition of the food safety acceptability of a food product. This was accomplished by plotting calibration curves of resonance in pm vs. the number of CFU/mL (Figure 10).

In quantitative analysis, the working interval is considered the set of values for which the test method has an adequate precision, trueness, and linearity. That is, it provides results with an acceptable level of uncertainty. As seen in Figure 10, the linear area was qualitatively assessed and confirmed by calculating the correlation coefficient. The qualitative evaluation of the straight line obtained was used to verify that the relationship between X and Y was linear and to ensure the linearity interval. As seen in the three curves obtained by graphical representation, we can visually confirm that the relationship between X and Y was linear. In addition, the correlation coefficient obtained and, therefore, the regression model obtained were acceptable in the three cases since r tended to 1.

The delimitation of the working interval for each calibration curve made it possible to estimate the lower limit or the approximate limit of quantification (LoQ) around 10^2^ CFU/mL to delimit the linear interval of the calibration. The limit of detection (LoD) to delimit the working interval was set around 10^1^–10^2^ CFU/mL. Additionally, the upper limit of quantification (ULOQ) imposed by the equipment or technique, reflected by the three curves, was the upper limit of the linear interval, around 10^8^ CFU/mL.

The results obtained by other authors [65,66,67] in the detection of *L. monocytogenes* in meats using direct PCR and post-enrichment PCR confirm that low concentrations of *L. monocytogenes* can only be detected using short enrichment followed by a PCR assay.

The immunosensor was as efficient as qPCR in the specific detection of the pathogens tested due to the IMS, which allowed a first binding of each species with a species-specific antibody, followed by the capture of the bacteria on the immunosensor. Detecting the antibodies used in the protein chip allowed the specificity of detection.

The results obtained in this study are comparable to those of an investigation in which immunosensors were shown to quantitatively detect *L. monocytogenes* from stationary-phase broth cultures using the EIS method, with a detection limit as low as 5.00 CFU/mL for *L. monocytogenes* [30].

The advantages of the immunosensor method over qPCR are that it does not require bacteria processing with lysis buffer and DNA purification kits as in the PCR analysis of *Listeria* spp. Another advantage of using specific antibodies for detection is that no extensive preliminary work is required, as is necessary in evaluating primer specificity for developing qPCR protocols.

## 4. Conclusions

This paper presents the validation of an alternative detection technique for *L. monocytogenes* in food samples based on a photonic biosensor. The biosensor was fabricated in silicon nitride using CMOS-compatible techniques such as electron beam lithography and RIE-ICP etching. The proposed biosensor comprises eight ring resonators, which were functionalized with different antibodies to detect *L. monocytogenes* on meat samples from the food industry in the frame of the Feder Bacterio Project.

The *L. monocytogenes*-specific polyclonal antibody has a greater capacity for binding to antigens and a wide range of concentrations. The *L. monocytogenes*-specific monoclonal antibody is more specific and has a more remarkable binding ability at low antigen concentrations. At very high concentrations of the antigen, no results were obtained. The result was proportional in terms of the absorbance as the signal saturated. The immobilization control results showed that the activated surfaces presented higher fluorescence intensity values than those of nonactivated surfaces, with statistically significant differences. No statistically significant differences were observed between the monoclonal and polyclonal antibody fluorescence values. The monoclonal antibody and the polyclonal antibody specific for *Listeria monocytogenes* were successfully immobilized on silicon nitride surfaces. However, polyclonal antibodies presented better immobilization results on the silicon nitride surfaces designed to develop the sensor.

The fabricated PICs were used in a preliminary validation to detect *L. monocytogenes* bacteria after the functionalization of the rings with specific antibodies and attaching a microfluidic layer on the PIC; the positive detection of several bacterial concentrations was achieved, demonstrating the excellent performance and feasibility of the proposed detection technique.

The traditional culture method requires a minimum of 5 days to determine whether the food is *Listeria*-free and, if not, 10 days to recognize whether *Listeria* spp. or *L. monocytogenes* is present. The biosensor developed in this research can be used as a diagnostic tool to monitor the safety of food products, such as raw beef, in just 4 h, thereby shortening the diagnosis of this microorganism. In addition, it permits an increasing number of samples to be examined in the industry to optimize food inspection and the food safety process.

## Figures and Tables

**Table 1:** Comparison of the results obtained by the immunosensor method with those obtained by each enrichment culture from different strains of *L. monocytogenes*, *L. innocua*, and replicas of the same batch of fresh meat samples analyzed.

Target Species/Sample *	Inmunosensor Response **	Count on Selective Agar (CFU/mL) ***
*L. monocytogenes* CECT933 04/2022	+	1
*L. monocytogenes* CECT911 04/2022	−	0
*L. monocytogenes* CECT936 04/2022	+	>10^8^
*L. monocytogenes* CECT936 05/2022	+	1.4 × 10^10^
*L. monocytogenes* CECT936 05/2022	+	4.1 × 109
*L. monocytogenes* CECT936 05/2022	+	3.5 × 10^8^
*L. monocytogenes* CECT936 05/2022	+	2.1 × 10^7^
*L. monocytogenes* CECT936 05/2022	+	2.1 × 10^7^
*L. monocytogenes* CECT936 06/2022	+	6.4 × 10^8^
*L. monocytogenes* CECT936 06/2022	+	1.7 × 10^8^
*L. monocytogenes* CECT936 07/2022	+	1.4 × 10^10^
*L. innocua* CECT910 04/2022	−	9.5 × 10^9^
*L. innocua* CECT910 04/2022	−	9.5 × 10^8^
*L. innocua* CECT910 05/2022	−	8 × 10^7^
*L. innocua* CECT910 06/2022	−	4.5 × 10^6^
*L. innocua* CECT910 07/2022	−	1 × 10^2^
*L. monocytogenes* M4 05/2022	+	9 × 10^10^
*L. monocytogenes* M4 05/2022	+	3.0 × 10^2^
*L. monocytogenes* M4 05/2022	+	4.0 × 10^6^
*L. monocytogenes* M4 06/2022	+	1.7 × 10^8^
*L. monocytogenes* M4 06/2022	+	8.4 × 10^7^
*L. monocytogenes* M4 07/2022	+	1 × 10^10^
*L. monocytogenes* M4 07/2022	+	1 × 10^2^
*L. monocytogenes* M4 07/2022	+	1 × 10^1^
Deep-frozen hamburger 04/2022	+	2 × 10^1^
Deep-frozen hamburger 04/2022	+	2 × 10^8^
Deep-frozen hamburger 04/2022	+	2 × 10^9^
Deep-frozen hamburger 04/2022	+	2 × 10^10^
Deep-frozen hamburger 05/2022	−	0
Deep-frozen hamburger 06/2022	+	6.5 × 10^5^
Deep-frozen hamburger 06/2022	+	6.5 × 10^6^
Deep-frozen hamburger 07/2022	+	6.5 × 10^10^
Deep-frozen hamburger 09/2022	+	<10^3^
Deep-frozen hamburger 10/2022	+	1.2 × 10^7^
Deep-frozen hamburger 11/2022	+	1.1 × 10^3^

* Replicates ordered according by the date of sampling inoculums strains and real food samples with presence of *L. monocytogenes*; ** + presence of *L. monocytogenes*, − absence of *L. monocytogenes*; *** *L. monocytogenes* contamination was estimated from first dilution of each sample at CFU/mL; *L. innocua* contamination was estimated from first dilution of each sample at CFU/mL. The corresponding CFU/mL of *L. monocytogenes* in the enrichment cultures was derived from the calibration curve shown in Figure 8.

## Data Availability

Not applicable.
